# Vertical Migration of Second-stage Juveniles of *Meloidogyne enterolobii* as Influenced by Temperature and Host

**DOI:** 10.2478/jofnem-2024-0012

**Published:** 2024-04-22

**Authors:** Ana Karina S. Oliveira, Elvira M. R. Pedrosa, Diego A. H. S. Leitão, Janete A. Brito, Ênio F. de F. Silva, Donald W. Dickson

**Affiliations:** Agricultural Engineering Department, Federal Rural University of Pernambuco, Recife, Pernambuco 52171-900, Brazil.; Soil, Water and Ecosystem Sciences Department, University of Florida, Gainesville, FL 32608, USA. (Current address); Entomology and Nematology Department, University of Florida, Gainesville, FL 32608, USA.; Florida Department of Agriculture and Consumer Services, Division of Plant Industry, Gainesville, FL 32608, USA.

**Keywords:** Behavior, *Meloidogyne enterolobii*, migration, *Solanum lycopersicum*, *Tagetes patula*

## Abstract

Infective second-stage juveniles (J2) of *Meloidogyne* spp. migrate towards host roots, which depends on several factors, including root exudates and soil temperature. Although *Meloidogyne enterolobii* is a highly virulent nematode that affects major agricultural crops worldwide, there is limited ecological data about it. The objective of this study was to determine the J2 migration pattern vertically in 14-cm long segmented soil columns towards tomato (*Solanum lycopersicum*) and marigold (*Tagetes patula*) roots, each grown at two soil temperatures (20 or 26ºC). Bottomless cups with tomatoes or marigolds were attached to the top of each column; cups with no plants were used as untreated controls. Juveniles (1,000/column) were injected into a hole located 1 cm from the bottom of each column. The apparatuses were placed in growth chambers at 20 or 26ºC, and J2 were allowed to migrate for 3, 6, 9, or 12 days after injection (DAI). At each harvest, J2 were extracted from each ring of the columns and counted to compare their distribution, and root systems were stained to observe root penetration. *M. enterolobii* migrated over 13 cm vertically 3 DAI regardless of temperature, even without plant stimuli. The vertical migration was greater at 26ºC, where 60% of active J2 were found at distances >13 cm at 12 DAI. Temperature did not affect root penetration. Overall, a greater number of J2 was observed in tomato roots, and root penetration increased over time.

*Meloidogyne enterolobii* (Yang and Eisenback, 1983) is one of the most damaging root-knot nematode (RKN) species because of its wide host range and ability to overcome RKN resistance genes in several agricultural crops (Carneiro et al., 2006; Brito et al., 2007; Cetintas et al., 2008; Kiewnick et al., 2009; Pinheiro et al., 2013; Gonçalves et al., 2014; Rosa et al., 2015). Since 2000, when the first outbreaks of *M. enterolobii* were observed in major guava-producing states in Brazil (Carneiro et al., 2001; Gomes et al., 2011; Pereira et al., 2009), reports on the occurrences of this species also damaging other economically important crops rapidly increased worldwide. Currently, *M. enterolobii* is considered the major pathogen and threat to guava production in tropical regions (Elling, 2013) and to sweet potato in the USA, where it has been reported to cause severe yield reductions and poor tuber quality (Rutter et al., 2019). As a result, *M. enterolobii* has been added to lists of pests recommended for regulation as quarantine organisms in the USA and Europe (Anonymous, 2017; Anonymous, 2018a, 2018b; EPPO, 2020).

After hatching from the eggs, second-stage juveniles (J2) of RKN migrate randomly through the porous spaces between soil particles in search of the roots of host plants (Abad et al., 2009; Bilgrami and Gaugler, 2004). Once a suitable host is found, it migrates coordinately toward its roots, penetrates root tips, moves within the cortex until finding an appropriate cell to establish a feeding site, and initiates parasitism (Elling, 2013; Warmerdam et al., 2018). During the host-finding phase, changes in environmental variables and soil attributes significantly influence the process of J2 migration (Carrillo and Hallem, 2015; Gallardo et al., 2015; Hunt et al., 2001; Wallace, 1968).

Temperature is a major factor for RKN J2 development, egg hatch, and movement (Curtis et al., 2009; Dávila-Negrón and Dickson, 2013; Leitão et al., 2021a). Prot and van Gundy (1981a) observed greater migration of *M. incognita* at temperatures between 18 and 22ºC, whereas Wallace (1966) noted that *M. javanica* migration was greater at 25ºC. The vertical migration of three individual populations of *M. hapla, M. chitwoodi* race 1, and *M. chitwoodi* race 2 was studied at 12, 18, and 24ºC; J2 of all three species were able to migrate greater distances at 18ºC (Pinkerton et al., 1987). These findings indicate that distinct RKN species migrate differently in specific temperature ranges.

In addition to temperature, J2 respond to root exudates released by host plants (Wang et al., 2018). Chemotaxis is the primary means by which J2 perceive chemical cues and start migrating coordinately towards host roots (Andaló et al., 2014; Rasmann et al., 2012; Reynolds, et al., 2011). Although several studies have evaluated the chemotactic response of *Meloidogyne* in agar plates, such bidimensional assays do not significantly represent the soil matrix tridimensional environment. Thus, soil column assays provide more realistic results to field conditions (Spence et al., 2008). Preferential migration of J2 towards good hosts has been observed (Dalzell et al., 2011; Dutta et al., 2011; Prot, 1976); however, *Meloidogyne* J2 can randomly migrate long distances even under plant-free conditions (Leitão et al., 2021b; Pinkerton et al., 1987; Oliveira et al., 2020). Despite the economic importance of *M. enterolobii* to agriculture and food security and the amount of information available focusing on this nematode species in the last 20 years, little information is available about its migratory behavior within different soils.

The objectives of this study were to determine i) the migratory behavior of J2 of *M. enterolobii* under host (tomato) and nonhost (marigold) plant stimuli; ii) whether J2 can migrate long distances even under host-free conditions; and iii) the effect of temperature on the migration and root penetration of J2.

## Materials and Methods

### Nematode Inoculum

An isolate of *M. enterolobii* (N01- 00514) obtained from the RKN collection, Division of Plant Industry – DPI/FDACS, Gainesville, FL, USA (Brito et al., 1994) was reared on tomato (*Solanum lycopersicum* ‘Cobra’) in a greenhouse at the University of Florida, Gainesville, FL, USA and used in this study. The J2 were obtained by extracting nematode eggs using 0.52% sodium hypochlorite (NaOCl) solution according to Hussey and Barker (1973), modified by Bonetti and Ferraz (1981). The egg suspension was then poured onto modified Baermann funnels at 27ºC containing 2-ply facial tissue paper (Rodríguez-Kábana and Pope, 1981). After the first 24 hr, the J2 collected were discarded to avoid using old J2 for the assays (Leitão et al., 2021b). Freshly hatched J2 were collected on a sieve with 25-µm openings daily for 4 days and stored at 4ºC until the beginning of the experiment.

### Seedlings of tomato and marigold

Seeds of tomato ‘Cobra’ and marigold (*Tagetes patula* ‘Petite’) were sown into vermiculite in plastic seedling trays, one seed per cell, and placed in a greenhouse (28 ± 3ºC, 90–95% relative humidity) for germination. Four-week-old seedlings of both plant species were used in migration columns after their root systems were thoroughly washed with water to remove vermiculite debris.

### Experimental apparatus

**Figure 1:**
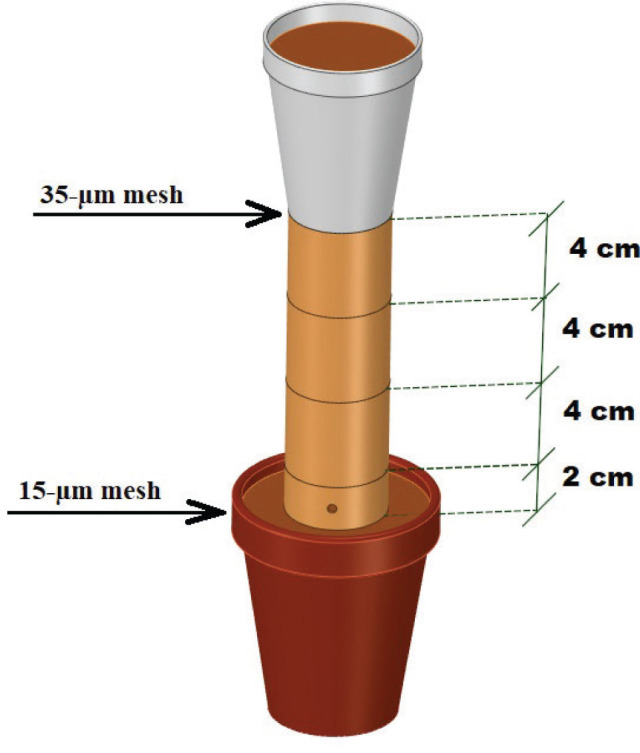
Diagram sketch of an experimental soil-filled column. Each column was comprised of PVC pipe cut into three 4-cm-long sections. Each section had an internal diameter of 4.4 cm and was taped tightly together. A 2-cm-long injection ring was placed at the bottom of each migration column with a hole through which nematode suspensions could be injected. A 15-µm mesh screen was attached to the bottom of each injection ring. A 300-cm^3^ Styrofoam cup with the bottom removed was taped to the top of each migration column. Each cup had a nylon mesh screen with 35-µm openings taped to the bottom. One-third of the columns had no plant, one-third contained tomato (*Solanum lycopersicum*), and one-third contained marigold (*Tagetes patula*) seedlings.

**Figure 2:**
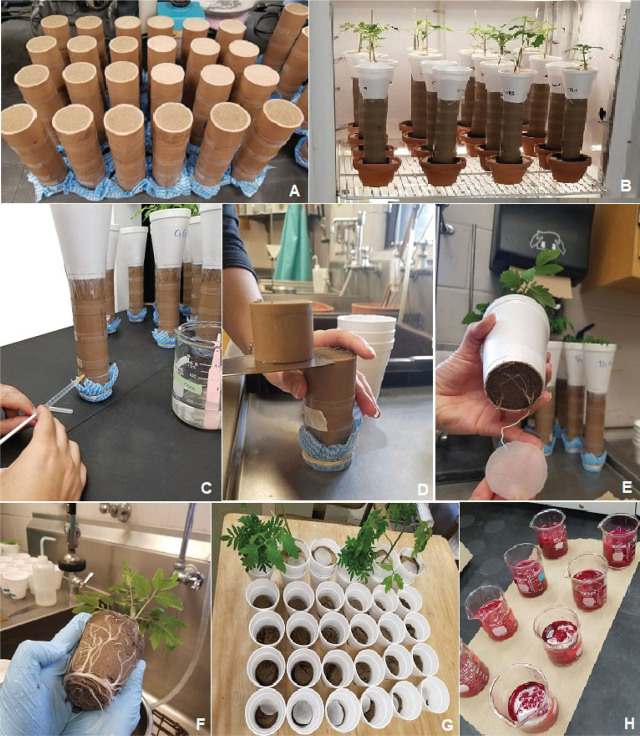
Experiment step-by-step. A: Soil columns filled with sandy soil; B: Styrofoam cups with either no plant, tomato, or marigold were attached to the columns and then transferred to separate growth chambers, each set at either 20 or 26ºC; C: Injection of suspension of second-stage juveniles of *Meloidogyne enterolobii* into the hole in the injection ring; D: Dismantling of each column was performed with a spatula to separate the rings; E: The 35-µm mesh screen prevented roots from growing into the columns; F: An example of a tomato plant root system at 12 DAI; G: Soil from each ring was placed in separate cups for centrifugal-flotation extraction of second-stage juveniles; H: Root staining with acid fuchsin was used for determining nematode penetration numbers.

The migration of *M. enterolobii* was evaluated in polyvinyl chloride (PVC) soil columns (Fig. 1) based on the specifications by Pinkerton et al. (1987). The columns consisted of a Styrofoam cup attached to three 4-cm long rings and one 2-cm long ring (inoculation ring) with a 4.4 cm internal diameter. A hole was drilled into the inoculation ring, 1 cm above its base, for the injection of J2. The rings were taped together to provide a total internal volume of 213 cm^3^. Each column was filled with heat (82ºC) pasteurized Lamellic Quartzipsamments (96% sand, 2% silt, and 2% clay, 0.27% OM) (Soil Survey Staff, 2010) collected from a peanut field in Levy County, FL, USA (Fig. 2A). The soil was compacted to provide 1.2 kg dm^−3^ bulk density to simulate field conditions (Table 1).

**Table 1:** Chemical attributes characterization of the soil used to fill the columns.

**Chemical attributes**	**Unit**	**Depth (m) 0.00–0.40**
pH	(1:2.5)	5.6
Extractable P	mg Kg^−1^	>135
Extractable K	mg Kg^−1^	13
Extractable Mg	mg Kg^−1^	0
Extractable Ca	mg Kg^−1^	164
Organic Matter	%	0.27

*Notes*: P: Phosphorus; K: Potassium; Mg: Magnesium; Ca: Calcium.

Bottomless Styrofoam cups with 300 g of soil were taped to the top of PVC columns and had a single tomato or marigold seedling transplanted or no seedling (control). To avoid roots from growing into the system, a nylon mesh with 35-μm openings was taped between the cups and the columns (Prot, 1976). In contrast, a nylon mesh with 15-μm openings (smaller than J2 body diameter) was attached to the bottom of the inoculation ring to prevent J2 from moving out of the ring (Pudasaini et al., 2007). The assembled columns were placed in environmental chambers under a completely randomized blocks design with four replicates, where half of the columns were kept at 20ºC and the other half at 26ºC, each with 16 hr light/8 hr dark photoperiod (Fig. 2B).

Soil water content was maintained at 10% by weight throughout the experiment by replacing the amount of water lost by evapotranspiration through daily top irrigation. To mitigate the effect of temperature oscillations while watering the columns, water bottles were kept inside each environmental chamber.

Approximately 1 ml of nematode suspension containing 1,000 ± 100 *M. enterolobii* J2 was injected into the inoculation hole (Fig. 2C). Columns were dismantled across each one of the five individual sections (Styrofoam cup, three middle rings, and inoculation ring) at 3, 6, 9, and 12 days after inoculation (DAI) (Fig. 2D). At each interval, one column of each stimulus treatment – tomato, marigold, and control – was randomly selected and plants were carefully uprooted. A total of 24 columns (three stimuli × four intervals × two temperatures) were dismantled per replicate, providing a grand total of 120 sections (24 columns × 5 sections) and 480 experimental units (120 sections × four replicates). Nylon meshes (35 and 15 µm) were checked under a stereomicroscope for trapped J2 at each sampling time and whether the 35-µm mesh screen prevented root growth into the columns (Fig. 2E). An example of a root system of a tomato seedling at 12 DAI is shown (Fig. 2F). J2 were extracted from the soil in each separate ring (Fig. 2G) by the centrifugal-flotation technique (Jenkins, 1964). The number of J2 retrieved from the soil of each ring and Styrofoam cup was counted and recorded. J2 were grouped as recovered (total number of nematodes) and active (J2 that showed movement regardless of intensity). During column sampling, the roots of tomatoes and marigolds were washed, weighed, and stained with acid fuchsin (Byrd et al., 1983) to compare root penetration between plants and over time (Fig. 2H). Additionally, fresh shoots and roots were weighed to record potential correlations with J2 migration.

### Statistical analysis

Nematode data were subjected to transformation √x + 0.05 before statistical analyses to homogenize variances. Repeated measures MANOVA was used to test the effects of temperature, plant stimuli, distance migrated, and time on the migration of J2 of *M. enterolobii*. A chi-square test was further performed on significant results to compare J2 distribution within the columns, and the least squared difference (LSD) mean comparison test was used for J2 inside roots at 5% probability. All the analyses were performed on the statistical software RStudio (RStudio, Boston, MA, 2015).

## Results

At each sampling period, no *M. enterolobii* J2 were found on the PVC ring walls or trapped within the meshes. There was an influence of temperature on recovered J2 (*P* < 0.0001), and there was an interaction between stimulus and distance (*P* < 0.05) and time and distance (*P* < 0.0001, Table 2). The migration of active J2 along the columns was influenced by the effects of each factor separately (*P* < 0.01), and there was a triple interaction among time, temperature, and distance (*P* < 0.05, Table 2).

**Table 2:** Repeated measure MANOVA summary of the effects of temperature, plant stimulus, section, and time on second-stage juveniles (J2) of *Meloidogyne enterolobii* vertical migration in PVC columns filled with sandy soil.

**Source**	**Recovered J2**					**Active J2**			
	
	**df**	**SS**	**MS**	**F**	***p*-value**	**SS**	**MS**	**F**	***p*-value**
Block	3	16.00	5.50	1.04	0.3755	86.91	28.97	8.37	<0.0001
Temperature (Temp)	1	129.20	129.20	24.62	<0.0001	194.18	194.18	56.13	<0.0001
Stimulus (Stim)	2	78.40	39.20	7.47	0.0007	47.94	23.97	6.93	0.0011
Section (Sec)	4	3884.40	971.10	184.97	<0.0001	628.20	157.05	45.40	<0.0001
Temp×Stim	2	10.80	5.40	1.02	0.3603	1.30	0.65	0.19	0.8276
Temp×Sec	4	4.40	1.10	0.20	0.9380	8.60	2.15	0.62	0.6469
Stim×Sec	8	96.00	12.00	2.29	0.0210	41.12	5.14	1.48	0.1614
Temp×Stim×Sec	8	78.40	9.80	1.86	0.0653	35.44	4.43	1.28	0.2526
Time	3	142.20	47.40	9.03	<0.0001	146.28	48.76	14.09	<0.0001
Time×Temp	3	3.00	1.00	0.19	0.9065	16.98	5.66	1.64	0.1805
Time×Stim	6	45.00	7.50	1.43	0.2021	15.00	2.50	0.72	0.6322
Time×Sec	12	730.80	60.90	11.60	<0.0001	493.68	41.14	11.89	<0.0001
Time×Temp×Stim	6	22.80	3.80	0.71	0.6383	15.72	2.62	0.76	0.6034
Time×Temp×Sec	12	55.20	4.60	0.88	0.5640	82.20	6.85	2.01	0.0228
Time×Stim×Sec	24	193.00	8.04	1.53	0.0547	106.80	4.45	1.29	0.1682
Time×Temp×Stim×Sec	24	64.80	2.70	0.52	0.9711	44.64	1.86	0.54	0.9652

Notes: df: degree of freedom; SS: Sum of squares; MS: Mean square.

**Figure 3:**
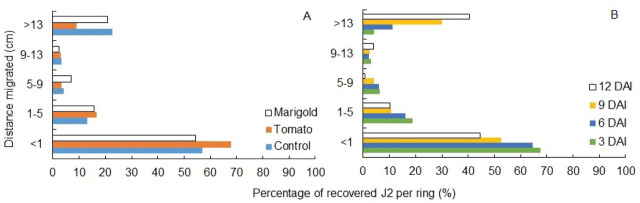
Distribution of recovered second-stage juveniles (J2) of *Meloidogyne enterolobii* extracted from PVC column sections each 4-cm long × 4.4-cm internal diameter. A. The percentages of second-stage juveniles (J2) migrating in columns containing either tomato, marigold, or no plant untreated control, where bars represent average data pooled from all temperatures and days after inoculation (DAI) (n = 32). B. The percentages of J2 migrating in columns sampled at four dates 3, 6, 9, and 12 DAI, where bars represent average data pooled from all temperatures and stimuli (n = 24). The bars among the different sampling periods are statistically different according to *X*^2^ test (*P* < 0.01).

More than 50% (range of 3053 to 3825 specimens) of recovered J2 were found in the inoculation ring regardless of plant stimuli (Fig. 3A). A migration tendency was observed along the columns; however, at distances greater than 13 cm, greater percentages of recovered J2 were observed for control and marigold columns (1523 and 1164 J2s, representing more than 20%). In contrast, only 9% (435) of recovered J2 were found at >13 cm under tomato (Fig. 3A). Regarding J2 migration over time, a steady reduction in the number of recovered J2 found in the inoculation ring occurred, as opposed to an increase in the number of J2 found at distances >13 cm. Almost 70% (3247) of recovered J2 were extracted from the inoculation ring at 3 DAI, whereas 44% (1502) were observed at 12 DAI (Fig. 3B). On the other hand, there was an increase in the percentage of J2 that were able to migrate more than 13 cm, where 4 (240), 11 (489), 30 (1025), and 40% (1368) were observed at 3, 6, 9, and 12 DAI, respectively (Fig. 3B).

**Figure 4:**
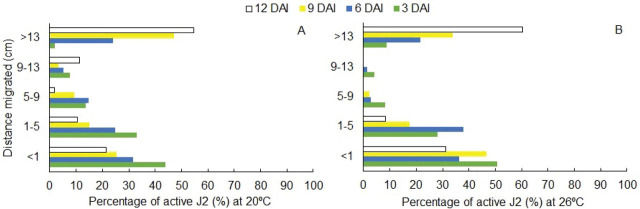
Distribution of active second-stage juveniles (J2) of *Meloidogyne enterolobii* in PVC soil columns (sections were 4-cm long and 4.4-cm internal diameter) over distance migrated (cm) and sampling time (3, 6, 9, and 12 days after inoculation [DAI]) at two different temperatures: 20ºC (A) and 26ºC (B). Each bar represents the average data pooled from all plant stimuli (n = 12). Distribution percentages were statistically different according to *X*^2^ test (*P* < 0.01).

At 3 DAI, irrespectively of temperature, the greater percentages of active J2 were found at the inoculation ring (Fig. 4). Nonetheless, some nematodes migrated 13 cm in the same period, a behavior that was even more evident at 26ºC, where around 9% (100) of active J2 was recovered near the rhizosphere of the plant (Fig. 4B). At 20ºC, active J2 percentage decreased with time in the inoculation ring, from 44% (713) to 22% (207) at 3 and 12 DAI, respectively (Fig. 4A); however, in the last section, there was an increase of active J2 over time at both temperatures (Fig. 4).

Active J2 between the second and fourth sections (1–13 cm) showed a different distribution pattern in relation to temperature. At 20ºC, the J2 were more evenly distributed over time, whereas at 26ºC, the number of active J2 recovered from 5 to 9 and 9 to 13 cm distances decreased (Fig. 4).

**Figure 5:**
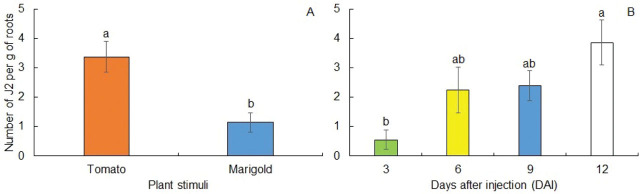
Penetration of second-stage juveniles (J2) of *Meloidogyne enterolobii* into tomato and marigold roots (A) and their rates over time for both plant species (B). Bars represent means of J2 compared by LSD test. Different letters indicate statistically different penetration rates at 5% of probability.

**Supplementary Figure 1:**
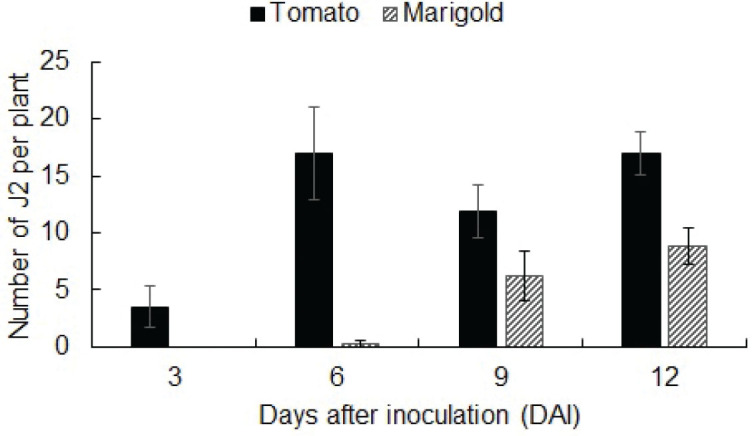
Penetration of second-stage juveniles (J2) of *Meloidogyne enterolobii* into tomato and marigold roots across 3, 6, 9, and 12 days after inoculation (DAI). Bars represent the average of J2 per plant for both temperatures (n=8), and error bars represent the standard errors of the mean. There was no interaction between stimulus and time; therefore, no statistical analysis was performed for this data.

The J2 of *M. enterolobii* inside roots after migrating through the column were influenced by stimulus and time (*P* < 0.0001, Table 3). The number of J2 that were able to penetrate roots was always higher in tomato roots throughout the experiment (Fig. 5A). Even though nematodes were found at distances greater than 13 cm at 3 DAI in the soil from the Styrofoam cups regardless of stimuli (Fig. 3), J2 were only observed inside tomato roots (Supplementary Fig. 1).

**Table 3:** Repeated measure MANOVA summary of second-stage juveniles (J2) of *Meloidogyne enterolobii* inside roots after migrating through sandy soil-filled PVC columns.

**Source**	**df**	**J2 inside roots**			

		**SS**	**MS**	**F**	***p*-value**
Block	3	2.96	0.99	2.99	0.0409
Temperature (Temp)	1	0.02	0.02	0.05	0.8333
Stimulus (Stim)	1	7.48	7.48	22.69	<0.0001
Temp×Stim	1	0.02	0.02	0.06	0.8067
Time	3	9.01	3.00	9.10	<0.0001
Time×Temp	3	0.22	0.07	0.22	0.8841
Time×Stim	3	2.74	0.91	2.76	0.0528
Time×Temp×Stim	3	0.07	0.02	0.07	0.9748

Notes: df: degree of freedom; SS: Sum of squares; MS: Mean square.

Shoot and root weight were significantly influenced by time (data not shown). Plant growth data suggest that the experimental period was not sufficient to cause damage to the plants due to *M. enterolobii* infection, except for root weight at 26ºC, which was nearly the same over time and even lower when compared to 20ºC at 12 DAI. This behavior might be due to the greater migration of J2 at 26ºC (Fig. 3). Since there was no significant interaction between stimuli and time, data from different DAI were combined in Fig. 5A, and data from both plant stimuli were combined in Fig. 5B. At 3 DAI, less than 1 J2/g of roots were able to penetrate plant roots. The number of J2 inside the roots of both plant species was higher at 12 DAI (P < 0.05) with approximately 4 J2/g of roots (Fig. 5B). It is important to mention that there was no penetration of *M. enterolobii* in *T. patula* at 3 DAI (Supplementary Fig. 1).

## Discussion

Nematode migration is defined as a coordinated movement of J2 towards host roots due to a gradient of root exudates (Bilgrami and Gaugler, 2004; Robinson and Perry, 2006); however, the literature on the behavior of J2 of *M. enterolobii* within the soil is scarce, especially in soil column migration assays.

Initially, most of the recovered J2 remained in the inoculation ring, but this decreased as the percentage of J2 moved away from the ring over time. Other researchers have noticed that the majority of injected J2 remained at or close to the inoculation ring in the short term (Francilino et al., 2017; Fujimoto et al., 2010; Nježić et al., 2014; Prot and van Gundy, 1981b; Pudasaini et al., 2007).

The presence of J2 at the top of the column at 3 DAI indicates that the texture of the Candler soil did not hinder nematode migration, which is directly related to soil pore space (Wallace, 1958a, 1958b). Nematodes can only migrate through pores wider than their body diameter (Wallace, 1968); thus, sandy soils most likely improve J2 migration because such soils are reported to have larger pore spacing (Rinaldi et al., 2014). The migration of J2 in soils with more than 30% clay content was negative (van Gundy, 1985).

The main approach for plant-parasitic nematodes to recognize plant hosts is chemotaxis (Reynolds et al., 2011). It is proposed that freshly hatched J2 migrate in relation to the attractiveness of root exudates (Ferraz and Brown, 2016).

After J2 hatch from eggs, they migrate within the soil without feeding (Wallace, 1966). They depend on the content of lipid reserves within their bodies to be motile until they find host roots (Das et al., 2011); therefore, the residence time in the soil is crucial for their viability and, consequently, their infection potential (Rocha et al., 2010, 2016). Reynolds et al. (2011) reported that nematodes tend to choose the shortest route to reach a good host plant, thereby saving energy reserves by remaining less time in soil.

The presence of a stimulus from a good host plant positively affected the vertical and horizontal migration of *M. javanica* (Prot, 1976) and *M. incognita* (Dalzell et al., 2011). On the other hand, vertical migration of *M. chitwoodi* occurred regardless of the presence of tomato plants in sandy-loam-filled columns (Pinkerton et al., 1987). The different results may be explained by the random root-knot nematode movement in the soil (Feltham et al., 2002) or varying traits among different *Meloidogyne* spp. The greater migration rate of *M*. *enterolobii* J2 observed at 26ºC during the first few days (3–6 DAI) may be attributed to the thermophilic trait of this species (Karssen et al., 2013). *M. hapla* and *M. chitwoodi*, which are considered cryophiles, showed a greater motility rate at 18ºC than at 24ºC (Pinkerton et al., 1987). In addition, J2 of *M. enterolobii* were found to be more mobile than *M. incognita* at 20ºC (Oliveira et al., 2020).

At 26ºC, the number of active J2 recovered from 5–9 and 9–13 cm decreased, suggesting a rapid movement of J2 towards plant roots. This behavior might suggest a rapid movement of the J2 towards the roots contained within the Styrofoam cup, which may be explained by an increased metabolism of *Meloidogyne* spp. J2, since they are poikilothermic organisms (Pudasaini et al., 2007), and promotion of root exudation changes on the cell membrane permeability and diffusion processes of the exudates (Neumann and Römheld, 2001) at higher temperatures.

Prot (1976) reported a high number of J2 of *M. javanica* inside tomato roots, 200 and 150 J2, in 25 and 50 cm columns, respectively, at 9 DAI. A 1.2-cm wide column was used, which might have favored J2 vertical migration. Columns with smaller diameters may not reflect field conditions since they favor the vertical migration of nematodes due to the small internal volume of the column and the lack of opportunity for lateral displacement of J2 (Spence et al., 2008). In fact, the higher percentage of *M. enterolobii* at the top of the control columns observed in our experiment might be a result of J2 random movement (Leitão et al., 2021a, 2021b), in addition to restricted lateral movement due to the volume limitation imposed by the PVC rings.

To our knowledge, there is no literature on the host status of *Tagetes* spp. to *M. enterolobii*. Marigold plants have been widely studied for suppressing plant-parasitic nematode population densities, especially *Meloidogyne* spp. (Kalaiselvam and Devaraj, 2011). There are records that certain types are resistant to *M. incognita* (Buena et al., 2008); however, data for *M. enterolobii* response is lacking. Rich et al. (2009) compiled information on the parasitism of different *Meloidogyne* spp. in several weed plants. No *M. enterolobii* parasitizing species were observed from the family Asteraceae, a botanical family to which *Tagetes* spp. belongs. Evaluating the host status of marigolds was not the objective of the present study; therefore, future studies with different species of marigolds must be performed to confirm their host status.

Extracts from parts of *Tagetes* spp. are used to manage RKN because of their repellent properties (Wang et al., 2018) that decrease J2 mobility of species such as *M. incognita*, *M. javanica,* and *M. paranaensis* (Munhoz et al., 2017). Marahatta et al. (2012) state that the suppressive effect is more evident when roots are actively developing. The results from our experiment indicate that *T. patula* may be useful in managing *M. enterolobii* since J2 take longer to penetrate its roots. The presence of J2 inside *T. patula* roots only at 9 and 12 DAI may indicate that it provides a less attractive stimulus to *M. enterolobii*.

In summary, *M. enterolobii* was able to migrate distances of more than 13 cm as soon as 3 DAI. Temperature influenced the vertical migration of *M. enterolobii*, which migrated faster at 26ºC, irrespective of the stimulus. Root penetration was always greater in tomato roots, whereas *T. patula* plants delayed the penetration of *M. enterolobii*.
